# A 35-bp Conserved Region Is Crucial for *Insl3* Promoter Activity in Mouse MA-10 Leydig Cells

**DOI:** 10.3390/ijms232315060

**Published:** 2022-12-01

**Authors:** Xavier C. Giner, Kenley Joule Pierre, Nicholas M. Robert, Jacques J. Tremblay

**Affiliations:** 1 Reproduction, Mother and Child Health, Room T3-67, CHU de Québec—Université Laval Research Centre, Québec, QC G1V 4G2, Canada; 2 Centre for Research in Reproduction, Development and Intergenerational Health, Department of Obstetrics, Gynecology and Reproduction, Faculty of Medicine, Université Laval, Québec, QC G1V 0A6, Canada

**Keywords:** Leydig cells, insulin-like 3, transcription, transcription factors, linker-scanning mutagenesis, DNA–protein interactions

## Abstract

The peptide hormone insulin-like 3 (INSL3) is produced almost exclusively by Leydig cells of the male gonad. INSL3 has several functions such as fetal testis descent and bone metabolism in adults. *Insl3* gene expression in Leydig cells is not hormonally regulated but rather is constitutively expressed. The regulatory region of the *Insl3* gene has been described in various species; moreover, functional studies have revealed that the *Insl3* promoter is regulated by various transcription factors that include the nuclear receptors AR, NUR77, COUP-TFII, LRH1, and SF1, as well as the Krüppel-like factor KLF6. However, these transcription factors are also found in several tissues that do not express *Insl3*, indicating that other, yet unidentified factors, must be involved to drive *Insl3* expression specifically in Leydig cells. Through a fine functional promoter analysis, we have identified a 35-bp region that is responsible for conferring 70% of the activity of the mouse *Insl3* promoter in Leydig cells. All tri- and dinucleotide mutations introduced dramatically reduced *Insl3* promoter activity, indicating that the entire 35-bp sequence is required. Nuclear proteins from MA-10 Leydig cells bound specifically to the 35-bp region. The 35-bp sequence contains GC- and GA-rich motifs as well as potential binding elements for members of the CREB, C/EBP, AP1, AP2, and NF-κB families. The *Insl3* promoter was indeed activated 2-fold by NF-κB p50 but not by other transcription factors tested. These results help to further define the regulation of *Insl3* gene transcription in Leydig cells.

## 1. Introduction

Leydig cells located in the testis produce testosterone and insulin-like 3 (INSL3), two hormones essential for male sex differentiation and reproductive function. More specifically, testosterone is required to masculinize the male embryo during fetal life, and postnatally to complete internal and external male organ development as well as for the initiation and maintenance of spermatogenesis [1]. INSL3 regulates the inguino-scrotal phase of testicular descent during fetal life [2,3] and bone metabolism in adults [4]. INSL3 has also been proposed to reduce germ cell apoptosis [5,6], modulate skeletal muscle metabolism and function [7], regulate motor and sensory brain functions [8], and contribute to corneal wound healing [9]. INSL3 is an exclusive marker for the differentiation and functional status of Leydig cells in the testis [10,11].

The *Insl3* gene is composed of two exons (219 and 404 bp) separated by a 739-bp intron. The *Insl3* gene is atypically located entirely within the last intron of the *Jak3* gene [12,13,14]. Consequently, the regulatory elements controlling *Insl3* gene expression in Leydig cells are believed to be located within a short promoter region of about 1000 bp. The genomic region corresponding to the *Insl3* promoter has been isolated from rat [13], mouse [12,15], human [16], dog [17], and pig [16]. Functional studies have identified several transcription factors regulating *Insl3* promoter activity in Leydig cells. These include the Krüppel-like factor KLF6 [18] as well as the nuclear receptors SF1 (Ad4BP, NR5A1) [13,17,19,20], LRH1 (NR5A2) [21], NUR77 (NGFI-B, NR4A1) [21,22], testosterone-activated androgen receptor (AR, NR3C4) [23,24], COUP-TFII (NR2F2) [25], and DAX1 (NR0B1) [19]. Transcriptional cooperation between COUP-TFII and SF1 [25,26], and between KLF6 and NUR77 and SF1 [18] on the *Insl3* promoter has also been reported. However, most of these transcription factors are also found in cell types that do not express the *Insl3* gene, such as Sertoli and adrenal cells, indicating that additional transcription factors are likely involved in directing *Insl3* expression in Leydig cells. In the present study, we have identified a 35-bp regulatory region bound by nuclear proteins that is essential for mouse *Insl3* promoter activity in MA-10 Leydig cells. 

## 2. Results

### 2.1. A 35-bp Region Is Responsible for 70% of Mouse Insl3 Promoter Activity

**Figure 1:**
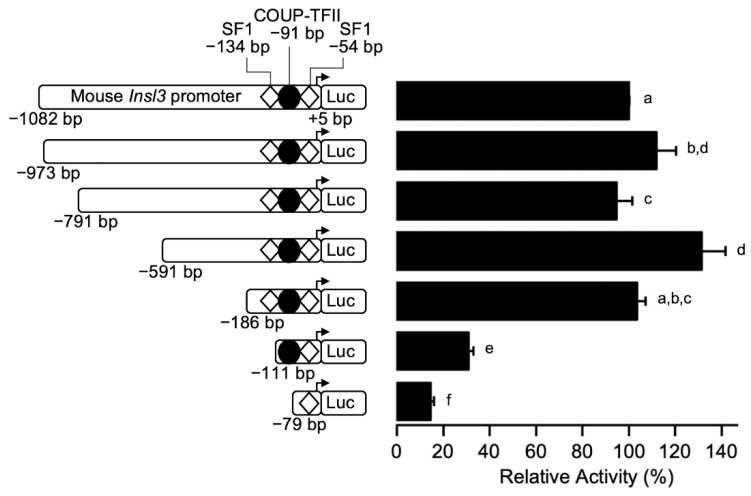
Identification of a critical regulatory region within the proximal mouse *Insl3* promoter. MA-10 Leydig cells were transfected with various 5’ deletion constructs of the mouse *Insl3* promoter; the 5′ end point of each construct is indicated on the left of the graph. The position of previously identified binding sites for SF1 (white diamonds) and COUP-TFII (black circle) is indicated. Results are shown as % Relative Activity (±SEM) relative to the activity of the −1132 bp reporter, which was set to 100%. Different letters indicate a statistically significant difference between groups (*p* < 0.05).

To identify the regions responsible for the activity of the mouse *Insl3* promoter in Leydig cells, several 5′ progressive deletions of the promoter were generated and transfected in MA-10 Leydig cells. The MA-10 cell line expresses the *Insl3* gene and has been validated as an appropriate model to study *Insl3* gene expression [20,22,27]. As shown in Figure 1, *Insl3* promoter deletions from −1082 bp to −186 bp had no significant effect on *Insl3* promoter activity. However, further deletion to −111 bp led to a 70% reduction in *Insl3* promoter activity (Figure 1). Finally, a further reduction in *Insl3* promoter activity to 15% was observed with a deletion to −79 bp, which is considered a minimal promoter (Figure 1). These data, therefore, identify a critical region located between −186 and −111 bp that is responsible for 70% of *Insl3* promoter activity. 

**Figure 2:**
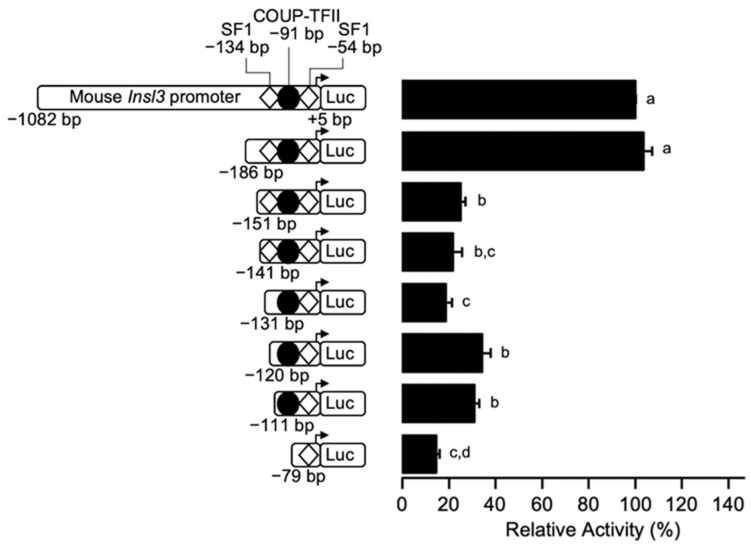
The region between −186 and −151 bp confers 70% of mouse *Insl3* promoter activity in Leydig cells. MA-10 Leydig cells were transfected with a series of fine 5’ deletion constructs of the mouse *Insl3* promoter; the 5′ end point of each construct is indicated on the left of the graph. The position of previously identified binding sites for SF1 (white diamonds) and COUP-TFII (black circle) is indicated. Results are shown as % Relative Activity (± SEM) relative to the activity of the −1132 bp reporter, which was set to 100%. Different letters indicate a statistically significant difference between groups (*p* < 0.05).

To more precisely locate the region conferring 70% of *Insl3* promoter activity, additional 5’ deletion constructs to −151, −141, −131, and −120 bp were generated and transfected in MA-10 Leydig cells. As shown in Figure 2, deletion of a 35-bp region between −186 and −151 bp led to a 70% reduction in *Insl3* promoter activity. Promoter activity remained similar with all other deletion constructs, except for the minimal −79 bp which was reduced to 15% (Figure 2). Together, these results indicate that a 35-bp region located between −186 and −151 bp is responsible for conferring 70% of activity to the mouse *Insl3* promoter in MA-10 Leydig cells.

### 2.2. Fine Mapping of the 35-bp Region Conferring 70% of Insl3 Promoter Activity

**Figure 3:**
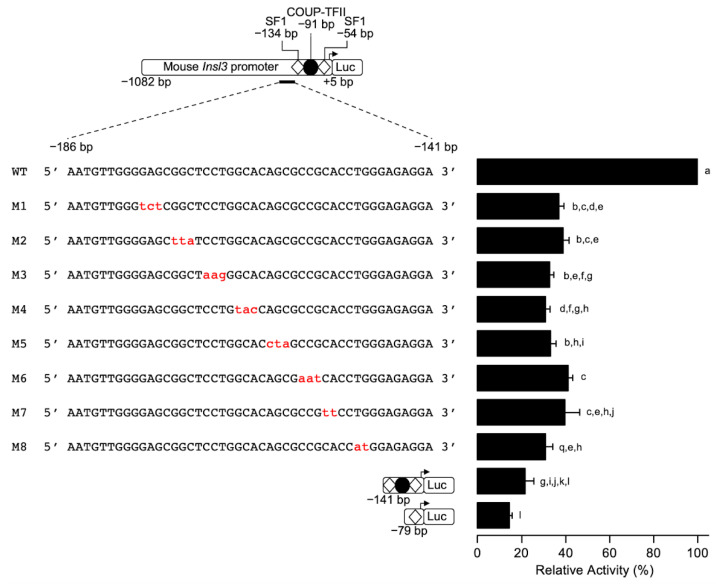
Fine mapping of the 35-bp region in the proximal mouse *Insl3* promoter. MA-10 Leydig cells were transfected with various −1132 bp mouse *Insl3* promoter constructs: a wild-type promoter and a series of trinucleotide or dinucleotide mutated constructs (M1 to M8; the mutations are in red lowercase). The position of previously identified binding sites for SF1 (white diamonds) and COUP-TFII (black circle) is indicated. Results are shown as % Relative Activity (± SEM) relative to the activity of the −1132 bp wild-type reporter, which was set to 100%. Different letters indicate a statistically significant difference between groups (*p* < 0.05).

To identify the sequences responsible for conferring 70% of mouse *Insl3* promoter activity within the −186 and −151 bp region, a linker-scanning approach was used. Eight sequential mutations were introduced in the 35-bp region in the context of the −1182 bp *Insl3* promoter (Figure 3) and the reporter constructs were transfected in MA-10 Leydig cells. As shown in Figure 3, all the mutations (M1 to M8) led to a loss of 60–65% in *Insl3* promoter activity compared to the wild type −1182 bp promoter. This indicates that the entire 35-bp region is necessary for full mouse *Insl3* promoter activity.

### 2.3. Binding of Nuclear Proteins to the 35-bp Regulatory Region

**Figure 4:**
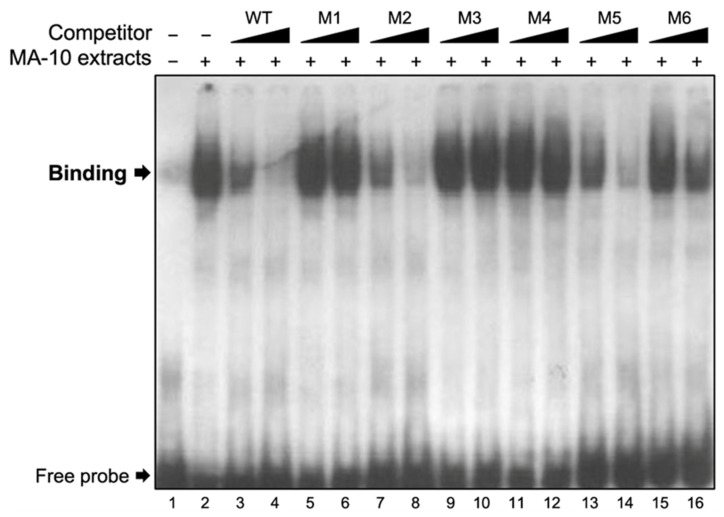
Nuclear proteins from MA-10 cells bind specifically to the 35-bp region. EMSA was used to determine the binding of nuclear extracts from MA-10 Leydig cells (MA-10 extracts) to a double-stranded ^32^P-labelled oligonucleotide corresponding to the 35-bp region (−186 to −151 bp) of the *Insl3* promoter. Protein binding was challenged by increasing concentrations (black triangles; molar excesses of 5× and 25×) of unlabelled oligonucleotides corresponding to the wild-type 35-bp region (WT) or oligonucleotides containing linker-scanning mutations (M1, M2, M3, M4, M5, M6; defined in Figure 3) in the 35-bp region.

The linker-scanning site-directed mutagenesis data (Figure 3) established that the full 35-bp region is important and that the activity of the *Insl3* promoter does not depend on any specific motif within this region. This suggests that binding of more than one transcription factor may be involved. A DNA–protein interaction approach (electromobility shift assay (EMSA)) was used to determine if protein(s) from Leydig cell nuclear extracts can bind to the 35-bp region. As shown in Figure 4, binding was detected (Figure 4, lane 2) that could be competed with increasing molar excess (5- and 25-fold) of unlabelled wild-type (WT) oligonucleotides (Figure 4, lanes 3 and 4). Due to the size of the band, it is possible that more than one protein binds to this region. To assess the specificity of the protein–DNA interaction, competition experiments were performed using unlabelled oligonucleotides (5× and 25× molar excess) containing the same mutations as those described in Figure 3 for promoter activity. As shown in Figure 4, oligonucleotides corresponding to mutants M1 (lanes 5 and 6), M3 (lanes 9 and 10), and M4 (lanes 11 and 12) were unable to compete the binding complex. Oligonucleotides corresponding to mutants M2 (Figure 4, lanes 7 and 8) and M5 (Figure 4, lanes 13 and 14) were as efficient as the WT oligonucleotide (Figure 4, lanes 3 and 4) at displacing the binding complex. On the other hand, oligonucleotides for mutants M6 only partially competed the binding complex (Figure 4, lanes 15 and 16). Together, these data indicate that protein(s) do bind to the 35-bp *Insl3* promoter region and that the nucleotides mutated in mutants M1, M3, M4, and to a lesser extent M6, are important for this binding.

### 2.4. Binding Assessment of Various Transcription Factors to the 35-bp Region 

**Figure 5:**
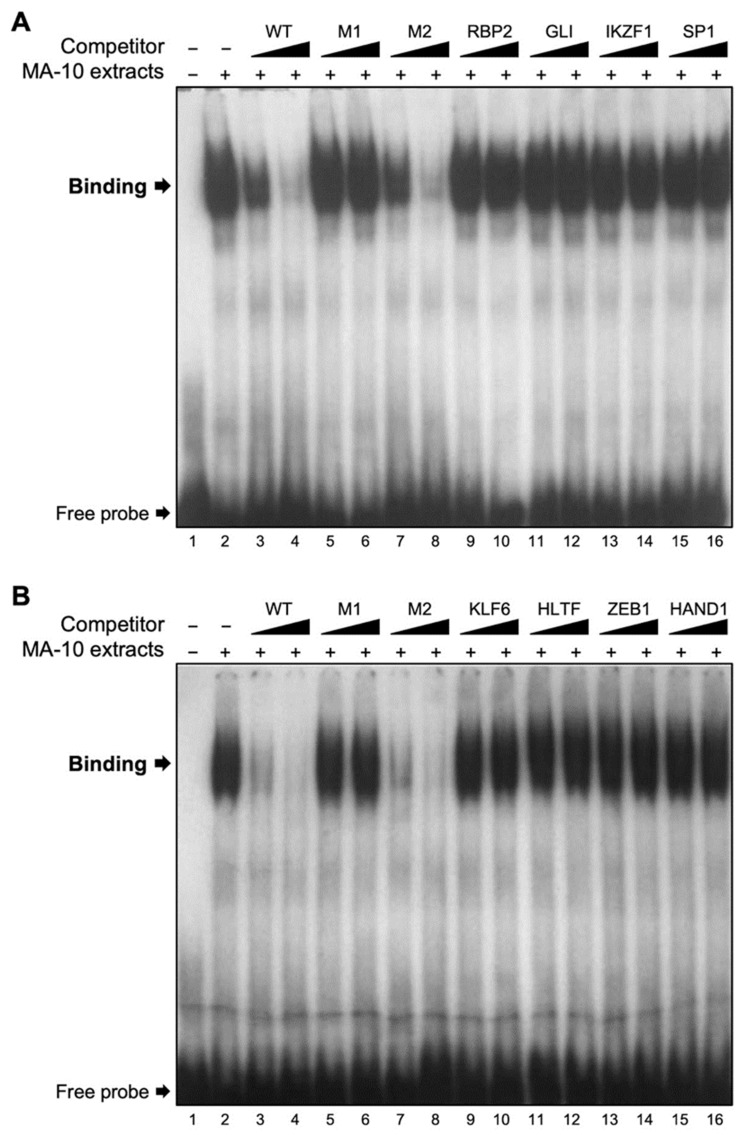
Assessment of potential transcription factors binding to the 35-bp region. EMSA was used to challenge the binding of nuclear protein(s) from MA-10 Leydig cells (MA-10 extracts) to a double-stranded ^32^P-labelled oligonucleotide corresponding to the 35-bp region (−186 to −151 bp) of the *Insl3* promoter. Protein binding was challenged by increasing concentrations (black triangles; molar excesses of 5× and 25×) of unlabelled oligonucleotides corresponding to the wild-type 35-bp region (WT), mutants M1 and M2, or oligonucleotides containing a consensus binding sequence for transcription factors (**A**) RBP2, GLI, IKZF1, SP1, and (**B**) KLF6, HLTF, ZEB1, HAND1.

The 35-bp nucleotide sequence (5′- AAT GTT GGG GAG CGG CTC CTG GCA CAG CGC CGC AC) contains potential binding sites for zinc finger-containing transcription factors known to bind GC- and GA-rich motifs. These include RBP2 (retinoblastoma-binding protein 2), GLI (GLI-Krüppel family zinc finger), IKZF1 (IKAROS family zinc finger 1), SP1 (specificity protein 1), KLF6 (Krüppel-like factor 6), HLTF (helicase-like transcription factor), ZEB1 (zinc finger E-box binding homeobox 1), and HAND1 (heart and neural crest derivatives-expressed protein 1). We, therefore, used oligonucleotides containing a consensus binding sequence for each of these transcription factors in EMSAs. As shown in Figure 5, binding of proteins from MA-10 Leydig cell nuclear extract was detected on the 35-bp region used as a probe (Figure 5A,B, lane 2). As expected, this binding was competed with unlabelled oligonucleotides corresponding the WT sequence and M1 mutant, but not M2 mutant (Figure 5A,B, lanes 3–8). Surprisingly, oligonucleotides containing a consensus binding site for RBP2, GLI, IKZP, SP1 (Figure 5A, lanes 9–16) and KLF6, HLTF, ZEB1, and HAND1 (Figure 5B, lanes 9–16) were unable to compete the binding complex. This indicates that these transcription factors do not bind to the 35-bp region of the mouse *Insl3* promoter.

### 2.5. NF-κB p50 Activates the Mouse Insl3 Promoter via the 35-bp Region 

**Figure 6:**
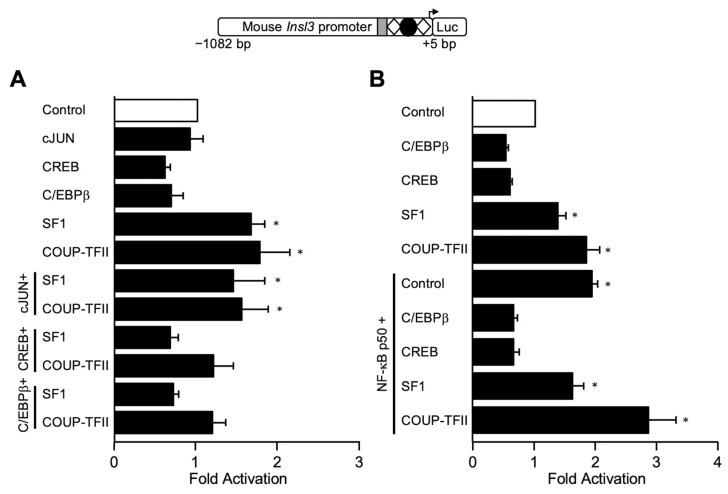
The mouse *Insl3* promoter is activated by NF-κB p50 but not CREB, cJUN, or C/EBPβ. MA-10 Leydig cells were transfected with a −1132 bp mouse *Insl3* promoter along with an empty expression vector (open bar) or expression vectors for cJUN, CREB, C/EBPβ, NF-κB p50, SF1, and COUP-TFII either alone or in combination as indicated in (**A**,**B**) (black bars). The position of previously identified binding sites for SF1 (white diamonds) and COUP-TFII (black circle) is indicated. The grey box represents the 35-bp sequence responsible for 70% of *Insl3* promoter activity. Results are shown as Fold Activation over control ± SEM. An asterisk (*) represents a statistically significant activation compared to control (empty expression vector, value set at 1, *p* < 0.05).

A closer analysis of the M1 sequence suggested that this might represent a potential binding site for NF-κB p50, or for members of the CREB or C/EBP families of transcription factors. In silico analysis of the sequences in mutants M3 and M4 predicts that they could be recognized by members of the AP1 and AP2 families. Several AP1 members are known to be present in Leydig cells, including cJUN, a well-characterized activator of several genes (reviewed in [28]). We, therefore, tested whether members of these families could activate the *Insl3* promoter in MA-10 Leydig cells. As shown in Figure 6A,B, the −1082 bp *Insl3* promoter was activated by SF1 and COUP-TFII as previously reported [13,17,19,20,25,26]. On the other hand, cJUN, CREB, and C/EBPβ failed to activate the *Insl3* promoter construct (Figure 6A,B). In the presence of NF-κB p50, a 2-fold activation of the −1082 bp *Insl3* promoter was observed (Figure 6B). Combining two transcription factors did not result in any functional cooperation (Figure 6A,B). However, in the presence of CREB or C/EBPβ, activation by the nuclear receptors SF1 and COUP-TFII (Figure 6A), and by NF-κB p50 (Figure 6B), was abolished, a phenomenon that occurs when transcription factors compete for a limited amount of common co-factors.

**Figure 7:**
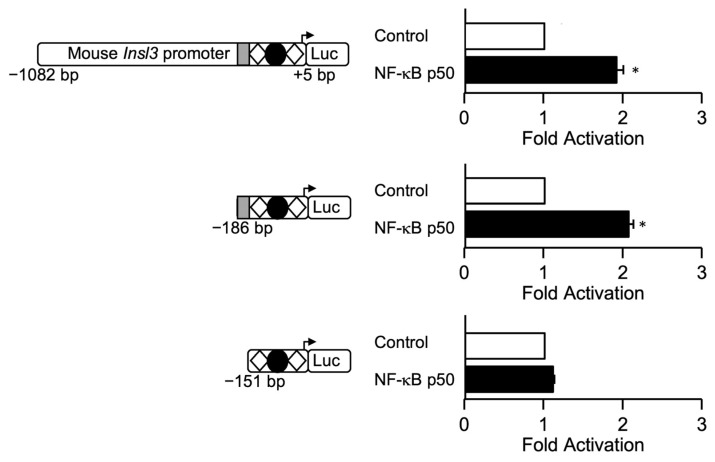
NF-κB p50-dependent activation of the mouse *Insl3* promoter requires the 35-bp region. MA-10 Leydig cells were transfected with three *Insl3* promoter constructs (−1132 bp, −186 bp, −151 bp) along with an empty expression vector (open bar) or an expression vector for NF-κB p50 (black bars). The position of previously identified binding sites for SF1 (white diamonds) and COUP-TFII (black circle) is indicated. The grey box represents the 35-bp sequence responsible for 70% of *Insl3* promoter activity. Results are shown as Fold Activation over control ± SEM. An asterisk (*) represents a statistically significant activation compared to control (empty expression vector, value set at 1, *p* < 0.05).

Next, to locate the NF-κB p50 responsive region, MA-10 Leydig cells were transfected with *Insl3* promoter deletion constructs. As shown in Figure 7, a deletion to −186 bp that retains the 35-bp region was still activated ~2-fold by NF-κB p50. However, a deletion construct to −151 bp that removes the 35-bp region was no longer activated by NF-κB p50 (Figure 7).

## 3. Discussion

The peptide hormone INSL3 produced by Leydig cells plays important roles in male reproductive development and function (reviewed in [29]). Although some transcription factors have been implicated in the regulation of *Insl3* gene expression in Leydig cells, most are also present in cells that do not express *Insl3* indicating that additional, yet unidentified transcription factors are also required to direct *Insl3* expression in Leydig cells. In the present study, we have located and characterized new regulatory elements important for *Insl3* promoter activity in Leydig cells. 

In our study, we made use of the mouse MA-10 Leydig cell line [30], which are immortalized cells corresponding to immature Leydig cells from the adult population [30,31]. Contrary to primary Leydig cells, MA-10 cells proliferate and contain an aberrant chromosome number [32]. Despite these issues, MA-10 cells nonetheless respond to hormonal stimulation, like primary Leydig cells, with an increase in steroid hormone production [30,33,34,35,36], indicating that the signalling cascades, kinases, and transcription factors required for this response are present in MA-10 Leydig cells. More relevant to our current work, MA-10 Leydig cells constitutively express *Insl3* [13,19,22,23,27,37,38,39] and have been validated as a suitable model to study *Insl3* gene transcription [27].

### 3.1. Identification of an Essential 35-bp Region within the Proximal Insl3 Promoter

**Figure 8:**
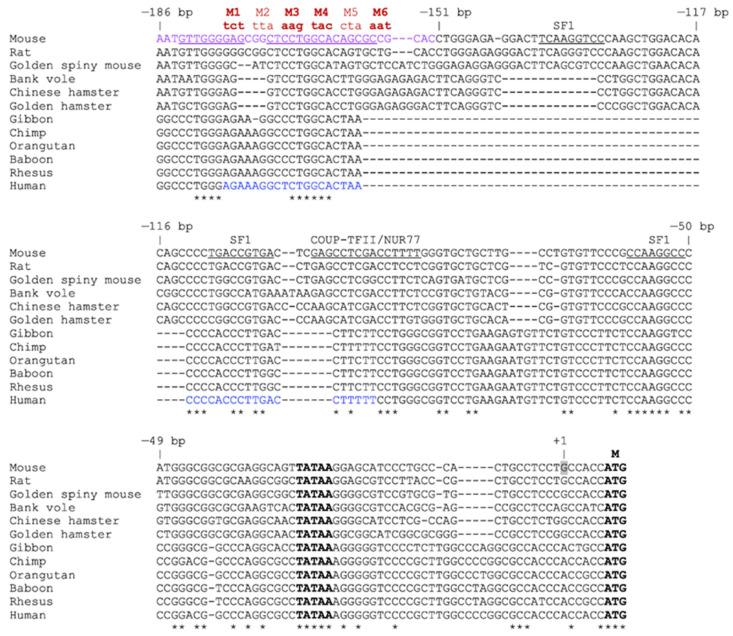
Alignment of the proximal *Insl3* gene promoter from different species. The sequences of the proximal *Insl3* promoter (−186 bp to the ATG of the mouse promoter) from various species indicated on the left were aligned using Clustal Omega multiple sequence alignment tool. The numbering corresponds to the position in the mouse *Insl3* promoter. The 35-bp region important for mouse *Insl3* promoter activity identified in this study is shown in purple. The mutations generated in the 35-bp region are shown in red (M1–M6); the bolded ones (M1, M3, M4, M6) were unable to compete the binding in EMSA. The underlined sequences correspond to potential binding sites for CREB, NF-κB, AP1, AP2, and C/EBP family members. The sequence shown in blue corresponds to an approximately 40-bp region (−132 and −93 bp) previously identified as important for the activity of the human *INSL3* promoter [18]. The positions of previously identified binding sites for transcription factors SF1, COUP-TFII, and NUR77 are shown. The TATA-box, transcription start site (+1), and ATG (M) are also indicated. An asterisk indicates a nucleotide conserved across all 12 species.

For the promoter functional assays, we used a genomic fragment of −1082 bp upstream of the mouse *Insl3* transcription start site. Because the entire *Insl3* gene is located within the last intron of the *Jak3* gene [12,13,14], the −1082 bp fragment corresponds to the longest sequence that can be used before entering the coding region of the *Jak3* gene. Our 5′ progressive deletion analysis of the mouse *Insl3* −1082 bp regulatory region revealed that deletion of up to −186 bp did not significantly affect *Insl3* promoter activity in MA-10 Leydig cells. This is similar to a previous study performed in another Leydig cell line, the MLTC-1 cells, where a construct of −188 bp retained all mouse *Insl3* promoter activity [19]. Another study also reported that a short proximal region of the mouse *Insl3* promoter contained within −157 bp was required for Leydig-specific transcription [20]. Deletion analysis of the human *INSL3* promoter also showed that truncation to −132 bp still maintained full promoter activity in both MA-10 and MLTC-1 Leydig cells but not non-steroidogenic cells [18]. Although the minimal required region appears shorter in the human promoter, the *Insl3* promoter from various rodents contains an additional ~50 bp compared to the *INSL3* promoter from primates, as revealed by the alignment of the *INSL3* promoter sequence from various species (Figure 8). Therefore, −132 bp in the human *INSL3* promoter is equivalent to −178 bp in the mouse *Insl3* promoter (Figure 8). Although the *Insl3* promoter has been isolated from rat, pig, and dog [13,16,17], a 5′ progressive promoter deletion approach to locate species-specific regulatory elements required for its activity in Leydig cells was not performed. 

Through fine promoter deletions, we identified a 35-bp region located between −186 and −151 bp that is essential for maximal *Insl3* promoter activity in Leydig cells. Site-directed mutagenesis in the context of the −1082 bp *Insl3* promoter further confirmed the importance of the entire 35-bp region since any trinucleotide or dinucleotide mutation within this region reduced promoter activity. This suggests that more than one transcription factor may bind to this region. The 35-bp region contains several GC- and GA-rich boxes, some of which have been conserved across species (Figure 8). In addition, a potential E-box motif (CAnnTG) for the binding of bHLH transcription factors is also present. The significant reduction in *Insl3* promoter activity that occurred when mutations were introduced in either half-site of this palindromic E-box motif (mutations M7 and M8) strongly supports the involvement of bHLH family members. In addition, mutations that target GC/GA-rich motifs reduce the activity of both the mouse (present work) and human [18] *INSL3* promoter in Leydig cells. This prompted us to assess whether proteins known to bind to GC/GA-rich motifs could bind to the 35-bp region of the mouse *Insl3* promoter. Although a protein complex from MA-10 Leydig cells specifically bound to the 35-bp region as identified by EMSA, competition experiments using several oligonucleotides containing binding sites for various transcription factors known to bind GC/GA-rich sequences such as RBP2, GLI, IKZF1, SP1, KLF6, HLTF, ZEB1, and HAND1, failed to displace the binding. This indicates that the protein complex binding to the 35-bp region does not contain members of these transcription factor families. 

### 3.2. Potential Transcription Factors Acting via the 35-bp Element

Our protein–DNA interaction assays indicated that the protein(s) responsible for the activity of the 35-bp region might not be zinc finger-containing transcription factors recognizing GC/GA-rich motifs. Of all the mutations introduced in the 35-bp region and tested by EMSA, mutants M1, M3, M4, and M6 failed displace the binding complex. This indicates that the integrity of these sequences is essential for protein binding, and thus, provides clues regarding the nature of the transcription factor that could bind to these sequences. 

In silico analysis of the sequence surrounding mutant M1 identified potential binding sites for members of the CREB, C/EBP, and NF-κB families of transcription factors, which are expressed in Leydig cells where they regulate gene expression [40,41,42,43,44,45]. However, when assayed in functional promoter assays, CREB and C/EBPβ failed to activate the *Insl3* promoter, either by themselves or in combination with other transcription factors. On the other hand, activity of the −1082 bp *Insl3* promoter was increased by 2-fold in the presence of NF-κB p50, supporting a role for this transcription factor in *Insl3* gene transcription in Leydig cells. Furthermore, the NF-κB p50-dependent activation of the *Insl3* promoter required the 35-bp region as a deletion construct lacking this region was no longer activated by NF-κB p50. Additional work such as performing EMSAs and competition assays using a consensus NF-κB p50 motif, using mutated *Insl3* promoter constructs, as well as testing other potential partners, would be needed to fully understand the mechanism of NF-κB p50 action in the regulation of *Insl3* promoter activity in Leydig cells.

Another sequence we identified within the 35-bp element as important for the binding of nuclear proteins and for the activity of the mouse *Insl3* promoter in MA-10 Leydig cells was the sequence 5′-TCCTGGCACA-3′ located between −170 and −161 bp. Mutation of this sequence (mutants M3 and M4) prevented protein binding and reduced *Insl3* promoter activity by 70%. In silico analysis of this sequence predicted that it could be recognized by members of the AP1 and AP2 families. Several AP1 members are known to be expressed in Leydig cells, including cJUN, a well-characterized activator of several genes (reviewed in [28]). AP2 factors are present in Leydig cells and regulate the activity of the luteinizing hormone receptor promoter [46]. Despite the fact that cJUN failed to activate the *Insl3* promoter in our assays, we cannot exclude the possibility that other AP1 or AP2 family members might contribute to *Insl3* promoter activity. At this time, the nature of the factor binding to this sequence remains to be established. This could be determined using a DNA–protein precipitation assay where a double-stranded oligonucleotide containing the sequence of interest is biotinylated and incubated with nuclear extracts. DNA-bound proteins are then isolated using avidin beads, eluted, and analyzed by LC-MS/MS. 

In conclusion, although our present work has identified a key 35-bp regulatory region, additional work is needed to fully decipher the transcription factors acting via this 35-bp element responsible for 70% of *Insl3* promoter activity in Leydig cells.

## 4. Materials and Methods

### 4.1. Plasmids

The mouse *Insl3* promoter constructs −1082 to +5 bp and −186 to +5 bp were previously described [22,25]. Mouse *Insl3* promoter deletions to −973 bp, −791 bp, −591 bp, −151 bp, −141 bp, −131 bp, −120 bp, −111 bp, and −79 bp were obtained by PCR using the −1082 bp *Insl3* promoter as a template, along the primers listed in Table 1. All promoter fragments were cloned into a modified pXP1 luciferase reporter plasmid [47,48].

Various trinucleotide and dinucleotide mutant constructs in the context of the −1082 bp reporter were generated using the QuikChange XL mutagenesis kit (Agilent Technologies Canada, Mississauga, ON, Canada), as recommended by the manufacturer, along with the oligonucleotides listed in Table 2 (only the sequence of the sense oligonucleotide is shown) where the mutations are in lowercase.

All the deletion and mutation reporter constructs were confirmed by sequencing (CHUQ Research Centre sequencing platform, Quebec City, QC, Canada). The following expression plasmids were obtained from different research groups: SF1/NR5A1 [49], cJUN [50], CREB [51], C/EBPβ [52], COUP-TFII/NR2F2 [53], and NF-κB p50 [54].

### 4.2. Cells Culture, Transfections, and Reporter Assays

Mouse MA-10 Leydig cells (ATCC, Manassas, VA, USA, Cat# CRL-3050, RRID:CVCL_D789) were grown in DMEM/F12 medium supplemented with 2.438 g/L sodium bicarbonate, 3.57 g/L HEPES, and 15% horse serum on gelatin-coated plates. Penicillin and streptomycin sulphate were added to the cell culture media to a final concentration of 50 mg/L, and cells were kept at 37 °C, 5% CO_2_ in a humidified incubator. MA-10 Leydig cells were validated by morphology and by quantifying steroidogenic output, as previously described [55,56,57,58,59,60,61,62]. MA-10 cells were transiently transfected using polyethylenimine hydrochloride (PEI) (Sigma-Aldrich Canada, Oakville, ON, Canada), as previously described [61,63,64], or the calcium phosphate co-precipitation method, as described in [22,35,48,65]. Briefly, MA-10 cells were transfected 24 h after plating at a density of 100,000 cells/well, by using 0.5 μg of *Insl3* promoter construct fused to the Firefly luciferase reporter gene, 20 ng of phRL-TK Renilla luciferase expression vector used as an internal control for transfection efficiency, and pSP64 as carrier DNA up to 1.5 μg/well. For transactivation assays, cells were transfected with 400 ng of the mouse *Insl3* −1082/+5 bp reporter vector along with 100 ng of an empty expression vector (pcDNA3.1 as control), or expression vectors for the various transcription factors (50 ng) individually (completed to 100 ng with the empty pcDNA3.1 expression vector to keep the total amount of expression vector to 100 ng), or the combination of transcription factors (50 ng each). The culture media were renewed 3 h before and 16 h after the transfection. Two days after the transfection, MA-10 Leydig cells were harvested and luciferase activities were measured using the Dual Luciferase Assay System (Promega Corp, Madison, WI, USA) and the Luminoskan Ascent luminometer (Thermo Scientific, Milford, MA, USA). The data reported represent the average of at least four experiments, using different DNA preparations, and each performed in duplicate.

### 4.3. Preparation of Nuclear Extracts

Nuclear extracts from MA-10 Leydig cells were prepared using the method described by Schreiber [66], with the following modifications: cells were rinsed with PBS-EDTA, harvested and pelleted by centrifugation for 45 s at 9000 RPM at 4 °C. The cells were resuspended in 700 µL of buffer A (HEPES 10 mM, EDTA 0.1 mM, EGTA 0.1 mM, DTT 1 mM, PMSF 0.5 mM, aprotinin 5 pg/mL, pepstatin 5 µg/mL, leupeptin 5 µg/mL) and incubated on ice for 15 min. Next, 50 µL of 10% Igepal was added and mixed vigorously for 10 s, followed by centrifugation for 30 sec at 13,000 RPM at 4 °C to pellet the nuclei. Nuclei were then incubated in 50 µL of buffer B (HEPES 20 mM, NaCl 400 mM, EDTA 1 mM, EGTA 1 mM, DTT 1 mM, PMSF 1 mM, aprotinin 5 µg/mL, pepstatin 5 pg/mL, leupeptin 5 µg/mL) with vigorous shaking for 45 min at 4 °C, followed by centrifugation for 5 min at 13,000 RPM at 4 °C. Protein concentrations were estimated using the standard Bradford assay (Bio-Rad Laboratories, Hercules, CA, USA). Nuclear proteins were stored at −80 °C until needed.

### 4.4. Electromobility Shift Assays

Electromobility shift assays (EMSAs) were performed as previously described [18,22,43,61,67,68,69,70,71]. Briefly, 5 µg of nuclear extracts from MA-10 Leydig cells were incubated in 20 µL of 4 mM Tris-HCl (pH 8.0), 24 mM KCl, 0.4 mM EDTA (pH 8.0), 0.4 mM dithiothreitol, 5 mM MgCl_2_, 100 ng BSA, 10% glycerol, and 500 ng poly(dI-dC) for 1 h on ice. A ^32^P-labeled 42-bp double-stranded oligonucleotide containing the 35 bp (−186/−151 bp) region of the mouse *Insl3* promoter was used as a probe (5′- GTT GGG GAG CGG CTC CTG GCA CAG CGC CGC ACC TGG GAG AGG -3′). Competition experiments were performed using 5× and 25× (molar excess) of unlabeled double-stranded oligonucleotides corresponding to the probe or harboring various mutations (shown in lowercase) in the 35-bp (−186/−151 bp) region of the mouse *Insl3* promoter (Table 2). Competitions were also performed using oligonucleotides corresponding to consensus binding sites for different transcription factors (Table 3).

### 4.5. Sequence Analysis

To identify binding sites for potential transcription factors, the 35-bp region of the mouse *Insl3* promoter was analyzed using the bioinformatic tools TFbind (https://tfbind.hgc.jp/, last accessed 10 October 2022) [80] and PROMO (PROMO version 3.0.2 using version 8.3 of TRANSFAC, last accessed 10 October 2022, http://alggen.lsi.upc.es/cgi-bin/promo_v3/promo/promoinit.cgi?dirDB=TF_8.3) [81,82]. Multiple sequence alignment was performed using the CLUSTAL Omega multiple sequence alignment tool (version 1.2.4, last accessed 12 October 2022, https://www.ebi.ac.uk/Tools/msa/clustalo/) [83].

### 4.6. Statistical Analysis

Statistical analyses were carried out using Statistics Calculators (Statistics Kingdom, Melbourne, Australia, November 2017, https://www.statskingdom.com/kruskal-wallis-calculator.html, accessed on 7 October 2022). To identify significant differences between multiple groups, statistical analyses were carried out using a nonparametric Kruskal–Wallis one-way ANOVA on ranks followed by a Mann–Whitney U test to detect differences between pairs. For all statistical analyses, *p* < 0.05 was considered significant.

## Figures and Tables

**Table 1:** Sequence of the primers used to generate the various mouse *Insl3* promoter deletion constructs.

−1082 bp	Forward	5′- GCG GAT CCT GGT TCC TAT GAT CTG GCT G -3′
−973 bp	Forward	5′- GCG GAT CCG AAT GGG GAT ATT AAA TAT GTG -3′
−791 bp	Forward	5′- GCG GAT CCC CCT TGC TCC CCT GAC TGT G -3′
−591 bp	Forward	5′- GCG GAT CCC TGG GAG AGT AGA GGT CTT G -3′
−186 bp	Forward	5′- CGG GAT CCA ATG TTG GGG AGC GGC TCC TG -3′
−151 bp	Forward	5′- GCG GAT CCC TGG GAG AGG ACT TCA AGG T -3′
−141 bp	Forward	5′- GCG GAT CCA CTT CAA GGT CCC AAG CTG G -3′
−131 bp	Forward	5′- GCG GAT CCC CCA AGC TGG ACA CAC AGC C -3′
−120 bp	Forward	5′- GGG GAT CCA CAC AGC CCC TGA CCG TG -3′
−111 bp	Forward	5′- GCG GAT CCC CTG ACC GTG ACT CGA GCC T -3′
−79 bp	Forward	5′- GGG GAT CCT GCT GCT TGC CTG TGT TC -3′
+5 bp	Reverse	5′- GGG GTA CCG TGG CAG GAG GCA GTG GGC AG -3′

The cloning sites are underlined: BamHI for the forward primers and KpnI for the reverse primer.

**Table 2:** Sequence of the oligonucleotides used to generate the various mouse *Insl3* promoter mutant constructs.

M1	5′- CTT GTT TTA AAT GTT GGG tct CGG CTC CTG GCA CAG CGC -3′
M2	5′- GTT TTA AAT GTT GGG GAG Ctt aTC CTG GCA CAG CGC CGC ACC -3′
M3	5′- AAA TGT TGG GGA GCG GCT aag GGC ACA GCG CCG CAC CTG -3′
M4	5′- GTT GGG GAG CGG CTC CTG tac CAG CGC CGC ACC TGG GAG -3′
M5	5′- GGG AGC GGC TCC TGG CAC cta GCC GCA CCT GGG AGA GGA -3′
M6	5′- GCG GCT CCT GGC ACA GCG aat CAC CTG GGA GAG GAC TTC -3′
M7	5′- CTG GCA CAG CGC CGC ACC atG GAG AGG ACT TCA AG -3′
M8	5′- CTG GCA CAG CGC CG ttC CTG GGA GAG GAC TTC AAG -3′

Only the sequence of the sense oligonucleotides is shown. Mutated nucleotides are in lowercase.

**Table 3:** Sequence of oligonucleotides corresponding to consensus binding sites for different transcription factors used in EMSA assays.

Transcription Factor	Sense Oligonucleotide	Reference
RBP2	5′- GGG CTC CCG CCC CAC GAA AAG -3′	[72]
GLI	5′- CGT CTT GGG TGG TCC ACG -3′	[73]
IKZF1	5′- TCA GCT TTT GGG AAT GTA TTC CCT GTCA -3′	[74]
SP1	5′- CGG CGC AGG GCG GGG CGG GGC GAG -3′	[75]
KLF6	5′- CCG AGG CCA CAC CCT ACT CTC TGA TAG TTC -3′	[76]
HLTF	5′- TTG ATT GAC ATA TA C CAG GAG ATA GA -3′	[77]
ZEB1	5′- GTG CAC AGT GCA AAG GTG GGG CGG CAG -3′	[78]
HAND1	5′- CAA CCA CAA TGG CGT CGT CTG GCA TTT TT -3′	[79]

Only the sequence of sense oligonucleotide is shown.

## Data Availability

All data generated or analyzed during this study are included in this article.
